# Surfactant-free production of biomimetic giant unilamellar vesicles using PDMS-based microfluidics

**DOI:** 10.1038/s42004-021-00530-1

**Published:** 2021-06-29

**Authors:** Naresh Yandrapalli, Julien Petit, Oliver Bäumchen, Tom Robinson

**Affiliations:** 1 Max Planck Institute of Colloids and Interfaces, Department of Theory and Bio-Systems, Potsdam, Germany; 2 Max Planck Institute for Dynamics and Self-Organization, Göttingen, Germany; 3 Experimental Physics V, University of Bayreuth, Bayreuth, Germany

**Keywords:** Membrane lipids, Lab-on-a-chip, Microfluidics, Bioanalytical chemistry

## Abstract

Microfluidic production of giant lipid vesicles presents a paradigm-shift in the development of artificial cells. While production is high-throughput and the lipid vesicles are mono-disperse compared to bulk methods, current technologies rely heavily on the addition of additives such as surfactants, glycerol and even ethanol. Here we present a microfluidic method for producing biomimetic surfactant-free and additive-free giant unilamellar vesicles. The versatile design allows for the production of vesicle sizes ranging anywhere from ~10 to 130 µm with either neutral or charged lipids, and in physiological buffer conditions. Purity, functionality, and stability of the membranes are validated by lipid diffusion, protein incorporation, and leakage assays. Usability as artificial cells is demonstrated by increasing their complexity, i.e., by encapsulating plasmids, smaller liposomes, mammalian cells, and microspheres. This robust method capable of creating truly biomimetic artificial cells in high-throughput will prove valuable for bottom-up synthetic biology and the understanding of membrane function.

## Abstract (web summary)

The production of giant unilamellar vesicles by microfluidics commonly involves additives, which may interfere with the resultant membrane properties. Here pure lipid GUVs are prepared which exclude residual surfactants and other additives.

## Introduction

Lipid-based vesicles have grown in popularity both in basic research and in application-oriented sciences, especially in pharmaceutics and cosmetics^1^. Lipids are not only biocompatible but are also the building blocks of life-forming vesicular structures, i.e., cells. While applications of lipid vesicles in the field of health care are advancing in the form of nanometer-sized liposomes^2^, their usability in understanding the evolution of cells and their various biochemical and physical pathways has hit a roadblock due to the lack of appropriate methods to form truly biomimetic cellular models, i.e., artificial cells. A cell mimic should fulfill the basic requirements of being lipid-based, vesicular in structure, and encapsulating the desired biomolecules such as enzymes, DNA, and even smaller vesicles as artificial organelles^3^. The latter being an essential step in the emerging and accelerating field of bottom-up synthetic biology^4,5^. Currently, the most common methods include the well-established electroformation and spontaneous swelling to produce giant unilamellar vesicles (GUVs)^6^. However, the limitations of these methods prevent high and uniform encapsulation of large and charged biomolecules. Although there are reports of encapsulating biomolecules in GUVs using swelling-based techniques, the reliability and reproducibility are low^7,8^.

**Fig. 1:**
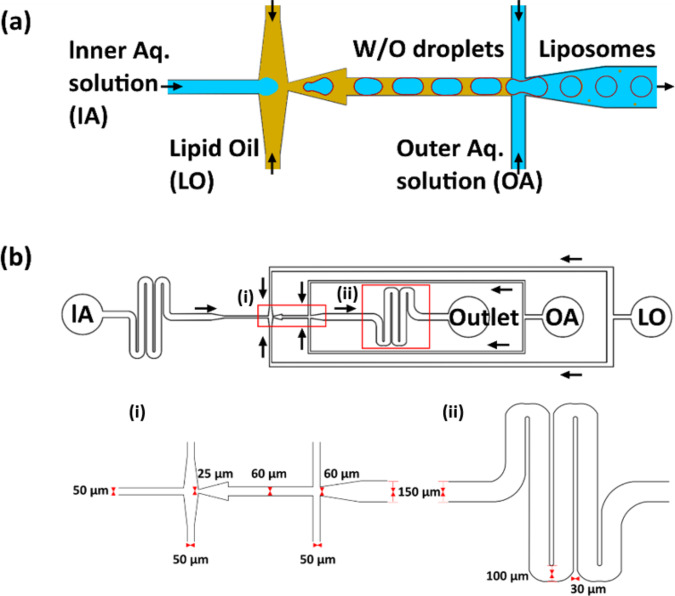
Microfluidic device design. **a** Schematic representation of liposome production using double a cross-junction design and **b** design of the microfluidic chip used in this study with details on various inlets and the flow directions (black arrows) from various channels. Insets show (i) the dimensions of the two cross-junctions (ii) and the second serpentine module after the junctions.

In the past few years, our group and many other researchers have turned towards emulsion-based technologies to overcome these issues and precisely control the uniformity of the encapsulates^9–13^. In our previous work, we showed that the inverted emulsion method can be a straightforward and reliable technique to produce basic models for cells, albeit with some drawbacks such as lack of size control, low throughput, and its dependency on the density of the solutions used^9^. Interestingly, all of the aforementioned drawbacks can be mitigated by implementing microfluidics to make lipid-based vesicles^14–18^. This involves microdroplet technology to create double emulsions of water-in-oil-in-water (W/O/W) with lipids in the oil phase. Typically, the production of lipid vesicles using microfluidics (see Fig. 1a) involves an inner aqueous solution (IA) that is sheared by an oil phase containing lipids (LO). This results in the formation of water-in-oil (W/O) single emulsions whose interface is assembled with a monolayer of lipids, thanks to their amphiphilic nature. The single emulsion is further sheared into droplets by an outer aqueous solution (OA) to form W/O/W double emulsions. Lipids present in the oil phase assemble along both the interfaces as the oil de-wets or is extracted to form liposomes. Based on the type of oil employed in solubilizing the lipids, the dewetting or extraction process varies from overnight incubation^19^ to as little as 5 min^14^. However, a major question remains regarding the biomimetic properties of the lipid vesicles due to the usage of surfactants and additives in both the aqueous phases^14,15,19^. For example, tri-block co-polymers made of poly(propylene)-co-poly(ethylene glycol)-co-poly(propylene) are extensively used to stabilize the liposome formation^16,19^. The copolymer works by incorporating itself, more specifically, the poly(propylene) chains, into the hydrophobic region of the lipid membrane, thus altering the biophysical properties of the membrane^20–23^. Additives such as glycerol and poly(vinyl alcohol) (PVA) used to improve the viscosity of the OA and IA phases for better manipulation of fluid flow, size control and emulsion stability, also affect the membrane properties^14,24^. It is well understood that as much as 10 wt% glycerol can completely saturate lipid head groups in a membrane which can cause crosslinking between adjacent phosphates and has been found to alter the diffusion of lipids^25–27^. During the production of microfluidic liposomes as much as 15 wt% glycerol is being employed^14,19^. Furthermore, PVA polymer chains can incorporate themselves across the bilayer and in some cases enhance protein aggregation^27,28^. Many studies also employ the use of ethanol as an additive which is well-known to alter the properties of membranes, hampering their use as cell mimics^15,17,29^. In light of these potential caveats, it is important to substantially advance existing microfluidic technologies and to be able to produce lipid vesicles that can encapsulate large biomolecules in high-throughput and yet, remain biomimetic. Such biomimetic lipid vesicles can potentially be used to progress the development of the long-envisioned concept of an artificial cell^4^.

In this work, a high-throughput microfluidic device is described that can produce said biomimetic lipid vesicles free from surfactants and additives with ease. Monodisperse GUVs with tunable sizes and with uniform encapsulation are shown. Importantly, we provided evidence of minimal to non-traceable levels of oil remaining in the final membranes by tuning the fluid flow rates. We demonstrate the flexibility of the method by creating membranes with neutral and charged lipids, as well as formation in a variety of environments - both physiological buffers and pure water. Finally, membrane functionality and the capability of encapsulating a wide range of biomolecules within the GUVs are demonstrated.

## Results

### Microfluidic design and GUV production

**Fig. 2:**
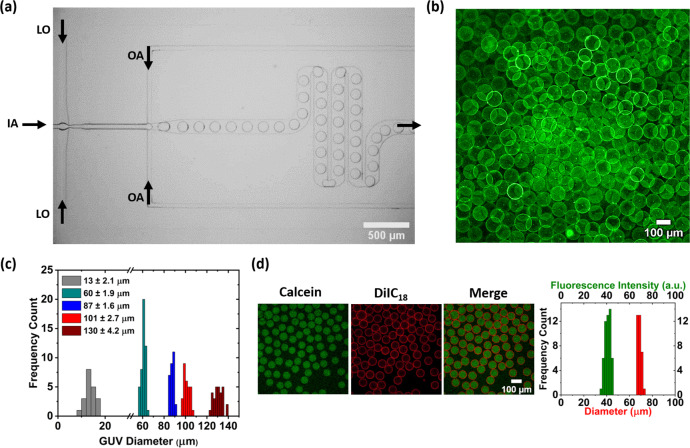
Production of monodisperse additive-free pure-lipid liposomes. **a** The production process using the microfluidic double emulsion device with 50 mbar at the IA (inner aqueous solution), 57 mbar at the OA (outer aqueous solution) and 44 mbar at the LO (lipid oil). **b** Confocal fluorescence image of GUVs with lipid composition POPC (99.5 mol%) and DiD (0.5 mol%) obtained with only water as the IA and OA. **c** Size distributions of the liposomes for different flow rate configurations, showing high size monodispersity over a wide range. **d** Confocal fluorescence images of negatively charged GUVs with lipid composition POPC (79.5 mol%), DOPS (20 mol%), DiIC_18_ (0.5 mol%) containing calcein (10 µM) together with the encapsulation efficiency (100%) and uniformity (RSD 6.2%) as well as their size homogeneity (67 ± 2 µm, RSD 2%).

A microfluidic design with a double cross-junction is implemented to produce liposomes in this study (Fig. 1b). The microfluidic chip is fabricated using PDMS-based techniques (see details under Methods) and plasma bonded to a glass coverslip (Supplementary Fig. 1a). Apart from the standard cross-junction to produce droplets, serpentine-shaped buffering channels are implemented in the design to reduce the risk of fluid backflow and act as fluidic resistors. One serpentine module is implemented before the first cross-junction where the IA is sheared into droplets by the LO and a second serpentine module is implemented after the second cross-junction (Fig. 1b). Without these modules, there is a marked decrease in flow stability and more flow discrepancies for longer periods. Indeed, the entire production process remains stable with no requirement of flow rate adjustments over a period of approximately one hour or until the reservoirs ran out of the solution (mostly the OA, due to the higher pressure-induced flow rate used). Apart from providing flow stability, the second serpentine module has constrictions (width is reduced from 150 to 100 µm) at every 180° turn, a total of four, providing increased fluidic velocities (Fig. 1b). These constrictions impart more shear force (Supplementary Fig. 1b for computational fluid dynamic analysis) while squeezing the double emulsions for excess oil removal (Supplementary Fig. 2a)^14,30,31^. The intrinsic friction provided by the serpentine module induces fast advective transport of lipids towards the interfaces to form stable membranes^30^. Furthermore, the high lipid concentration in the LO substitutes the need for surfactant usage to stabilize the liposomes and also contributes to faster monolayer assembly at the interfaces. Figure 2a shows an example of liposomes produced by pumping MilliQ^®^ water as both the IA and OA and 1-palmitoyl-2-oleoyl-sn-glycero-3-phosphocholine (POPC) (5 mg/mL) in 1-octanol as LO. To achieve this, initially the surface-functionalized chip (see details in the section “Methods”) is wetted by first pumping OA through the channels, followed by LO and IA. This strategy prevents the oil from eroding the surface coating (i.e., hydrophilicity) of the OA-outlet channel (a layer-by-layer self-assembled polyelectrolyte). Note that the coating process employed in this method takes ~5 min using a standard vacuum pump and also at room temperature, making the entire fabrication and usage of the microfluidic chip simpler compared to other methodologies employed elsewhere which require higher temperatures, longer times, or multiple pumps^14,16,32–34^. The pre-coating step renders the OA-outlet channels hydrophilic which is essential for the formation of stable double emulsions and to avoid the rupture of subsequently formed GUVs on their way to the outlet of the microfluidic chip. The final microfluidic device is ready to use immediately or even after a week, and for prolonged periods of time (>5 h).

Upon the introduction of all three fluids into the chip, the flow rates are adjusted (via pressure) to produce liposomes of a specific diameter. In this case, the fluid flow of the LO is set to 44 mbar, while the IA to 50 mbar and the OA to 57 mbar of pressure (Supplementary Movie 1). The resultant double emulsion formation rate is typically 3.2 ± 0.6 kHz. The high pressure-induced flow rates implemented at the second cross-junction are necessary to shear the W/O droplets and form very thin shells of the oil-phase (W/O/W). The thinness of the shell equates to lower amounts of 1-octanol and thus, faster removal with the help of the shear module. The leftover oil residue (if any) easily dewets from the double emulsion (Supplementary Fig. 2b, c). The final lipid vesicles, produced with only water both inside and outside, and with a diameter of 120 ± 5 µm are shown in Fig. 2b. To the best of our knowledge, these are the first giant lipid vesicles produced with pure water both inside and outside exhibiting very high size homogeneity and yield. It is important to specify that no surfactants or additives are used in the production process in order to retain the biomimetic aspects of the resulting membranes. The lack of thickening agents like PVA or glycerol requires the design to contain narrow channels and fluidic resistors to allow for easy tuning of the vesicle sizes. The device allows for the production of vesicles not only with a narrow size distribution but with tunable diameters (at least an order of magnitude range) unlike previous studies on microfluidic vesicle production^14,24^. Figure 2c depicts the histograms of vesicles with diameters of 13 ± 2.1 µm with a relative standard deviation (RSD) of 15.8%, 60 ± 1.9 with RSD of 3.2%, 87 ± 1.6 with RSD of 1.8%, 101 ± 2.7 with RSD of 2.7% and 130 ± 4.2 µm with RSD 3.2%. Note that the RSD value of the smallest set of liposomes produced is approximately three times higher than the vesicles of larger diameter in size, which is most likely due to the very high pressure-induced flow rates that have to be employed to yield smaller diameters and therefore reduced control at the second cross-junction/vesicle forming junction. If need be, this can be avoided by employing high molecular weight polyethylene glycol in the outer aqueous solution. Furthermore, we have tested the possibility of producing negatively charged lipid vesicles using this microfluidic device. Negatively charged membranes provide an opportunity to study the interaction and assembly of many positively charged membrane proteins, as well as serving as bacterial membrane models. Figure 2d shows confocal images of GUVs containing 1,2-dioleoyl-sn-glycero-3-phospho-L-serine (DOPS) in their membranes. The homogeneity in the size of the charged vesicles produced (67 ± 2 µm with RSD of 2%) and the encapsulation uniformity (here calcein dye) suggests the versatility of the device compared to a standard technique like electroformation where it is not possible to efficiently produce charged lipid vesicles.

### Validation of oil-free lipid membranes

**Fig. 3:**
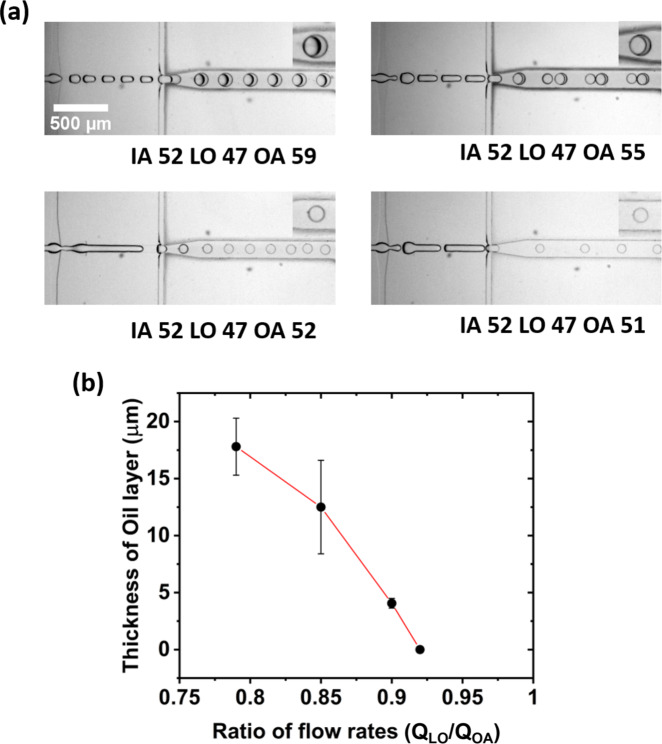
Manipulation of pressure-induced flow rates for reduced oil content and ultra-thin shells. **a** High-speed camera images of the microfluidic liposomal production process to reduce the oil thickness. Inserts: enlarged images of individual double emulsions. From the top left to the bottom right corner, the oil thickness has been reduced by gradually tuning the flow rates of the OA (outer aqueous solution). Pressures for each channel are given in mbar. **b** Plot showing the thickness of the oil layer that is reduced from 17 ± 2 to 12 ± 4 µm, 4 ± 0.5 µm and finally to optically non-detectable amounts by changing the flow rate (*Q*) ratios. Error bars are taken from the standard deviation of the mean (*n* > 15).

**Fig. 4:**
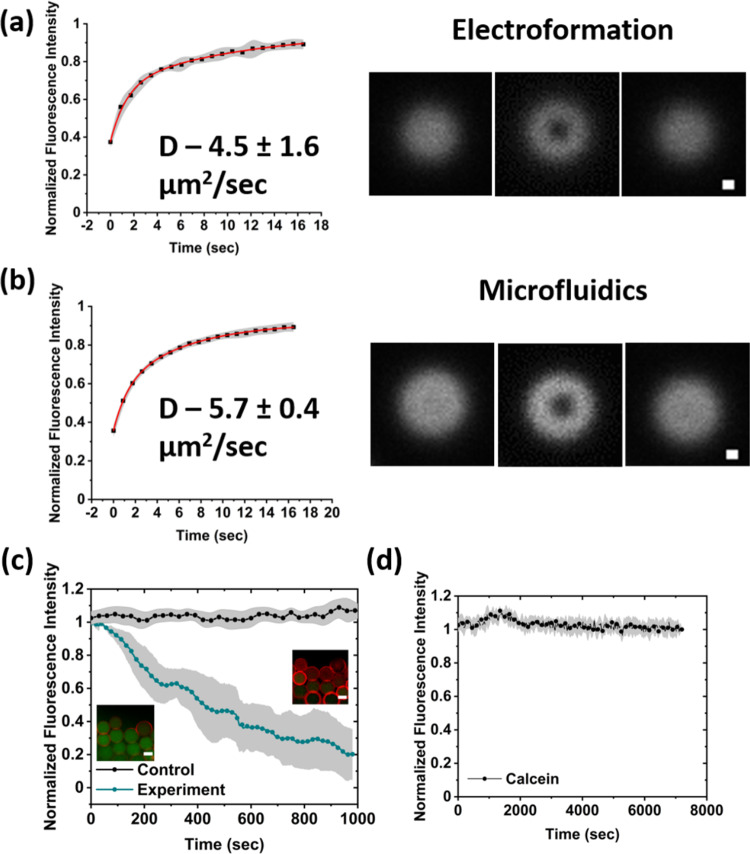
Purity and stability of the lipid membranes. Average fluorescence recovery curves (left) of at least 15 GUVs produced using **a** electroformation and **b** microfluidics along with the confocal cross-sections before (−2.5 s), during, and after (18 s) photobleaching (right). Scale bars: 3 µm. **c** A calcein dye-leakage assay using α-hemolysin protein insertion with a gradual decrease in mean luminal fluorescence intensity over time from vesicles with an average diameter of 60 µm (*n* > 10). Scale bars: 60 µm. **d** Plot showing the vesicle stability over time with no decrease in the mean calcein fluorescence intensity (*n* > 10). Error bars are taken from the standard deviation of the mean.

To ensure the biomimetic nature of the vesicles, it is not only important to avoid using surfactants and additives that can adversely affect the membrane’s biophysical properties, it is also important to quantify and validate the membrane properties systematically. In this respect, Fig. 3 shows the gradual decrease in the oil thickness present in the immediately formed double emulsions to finally achieve optically non-traceable levels. Figure 3a depicts bright-field images of the double emulsions produced using the same microfluidic device described earlier. The pressure-induced flow rates of the OA are gradually increased while the flow rates of the IA and LO are kept constant. By designing the OA channel width (50 µm) to be one-third the width of the outlet (150 µm), it is possible to shear W/O droplets with exceptional control to form W/O/W emulsions. Gradual increase in the pressure-induced flow rate of the OA channel makes a difference in the oil thickness from 17 ± 2 µm to negligible levels (Fig. 3b; Supplementary Movie 2). The physical mechanism at play in this transition is the rate of shearing and its dependency on high flow rates generated from high fluid pressures. A high fluid pressure of the continuous medium results in high shear rates. This can be easily understood for single emulsions such as W/O emulsions where increasing flow rates of the oil phase (continuous phase) result in decreasing water droplet size (dispersed phase). At the second cross-junction, the oil is the dispersed phase, thus increasing pressure (and hence flow rate) of the OA directly results in lower amounts of oil within the formed double emulsion. In addition to this, when the size of the W/O droplet is larger than the dimensions of the microfluidic channel (Supplementary Movie 2) but is not a continuous flow to the second cross-junction (as in Supplementary Movie 1), the first double emulsion created will have a thicker oil layer compared to the rest. However, it is possible to alter the thickness of the oil in the double emulsion based on the mechanism described earlier, and moreover, the oil eventually de-wets as shown in the Supplementary Fig. 2. However, since the optical resolution is well below that of the membrane thickness, we have performed lipid diffusion studies as well as a membrane poration assay to further ascertain the biomimetic nature of the liposomes. Lipid lateral diffusion is a signature characteristic that defines the purity of the membrane and can reveal impurities if the measured diffusion times differ from control values^26,35,36^. The diffusion times are determined by photobleaching the labeled lipids and observing the recovery of fluorescence in the bleached region over time. Using this information, it is possible to calculate the mobile fraction and hence the diffusion coefficient of the bleached lipid. Fluorescence recovery after photobleaching (FRAP) experiments are performed on liposomes containing 300 mOsm sucrose that are produced using both microfluidics and electroformation (see Fig. 4). The latter being the control as oils are not used. In both cases, the lipid composition is set as 99.5% POPC and 0.5% Liss Rhod DOPE. Figure 4a, b show the average fluorescence intensity recovery curves of the liposomes after photobleaching. Typical images of the bleached region of the liposomes along with snapshots before and after the exposure are also provided. From the fitting of the curves, the diffusion coefficients of Liss Rhod DOPE in electroformed and microfluidic liposomes were determined to be 4.5 ± 1.6 and 5.7 ± 0.4 µm^2^/s, respectively. These values suggest that the lipid lateral diffusion in the membrane is similar for both electroformed and our microfluidic liposomes. Furthermore, the diffusion coefficients observed in these measurements are in agreement with previous literature values^37^. A negative control was also performed on double emulsions with visible oil layers and resulted in a threefold increase in the diffusion coefficient due to the presence of free lipids in the oil (see Supplementary Fig. 3).

To further assess the quality of the liposomal lipid membrane produced in this work, a membrane protein-induced leakage assay was performed. Using α-hemolysin, a membrane-spanning heptamer, calcein dye leakage from the lumen of the liposomes into the outer solution was monitored. Microfluidic liposomes were produced with 20 µM calcein in the IA, placed on bovine serum albumin (BSA) coated coverslip, and flushed with α-hemolysin solution (final concentration of 2.5 µg/mL). The leakage profile of the calcein dye is plotted in Fig. 4c. Within a span of 10 min, calcein leakage from the lumen of the liposomes is observed while the fluorescence intensity of the control (no membrane pores) remained intact during the entire period. Not only does this finding demonstrate the functionality of the membranes but also their unilamellarity. In addition to this, the overall stability of the calcein-filled GUVs were analyzed for a period of 2 h. Figure 4d shows that the calcein intensity inside the lumen of the liposomes remained constant and the vesicles are stable when there is no external influence. Collectively all the studies performed: optically undetectable oil traces (Fig. 3), FRAP experiments to confirm the unhindered lipid lateral diffusion (Fig. 4a, b), membrane protein induced dye leakage assay (Fig. 4c), vesicle stability studies (Fig. 4d), and all without the use of surfactants or additives, supports the biomimetic pure-lipid nature of the liposomes produced with our microfluidic technique.

### Encapsulation toward complex artificial cells

**Fig. 5:**
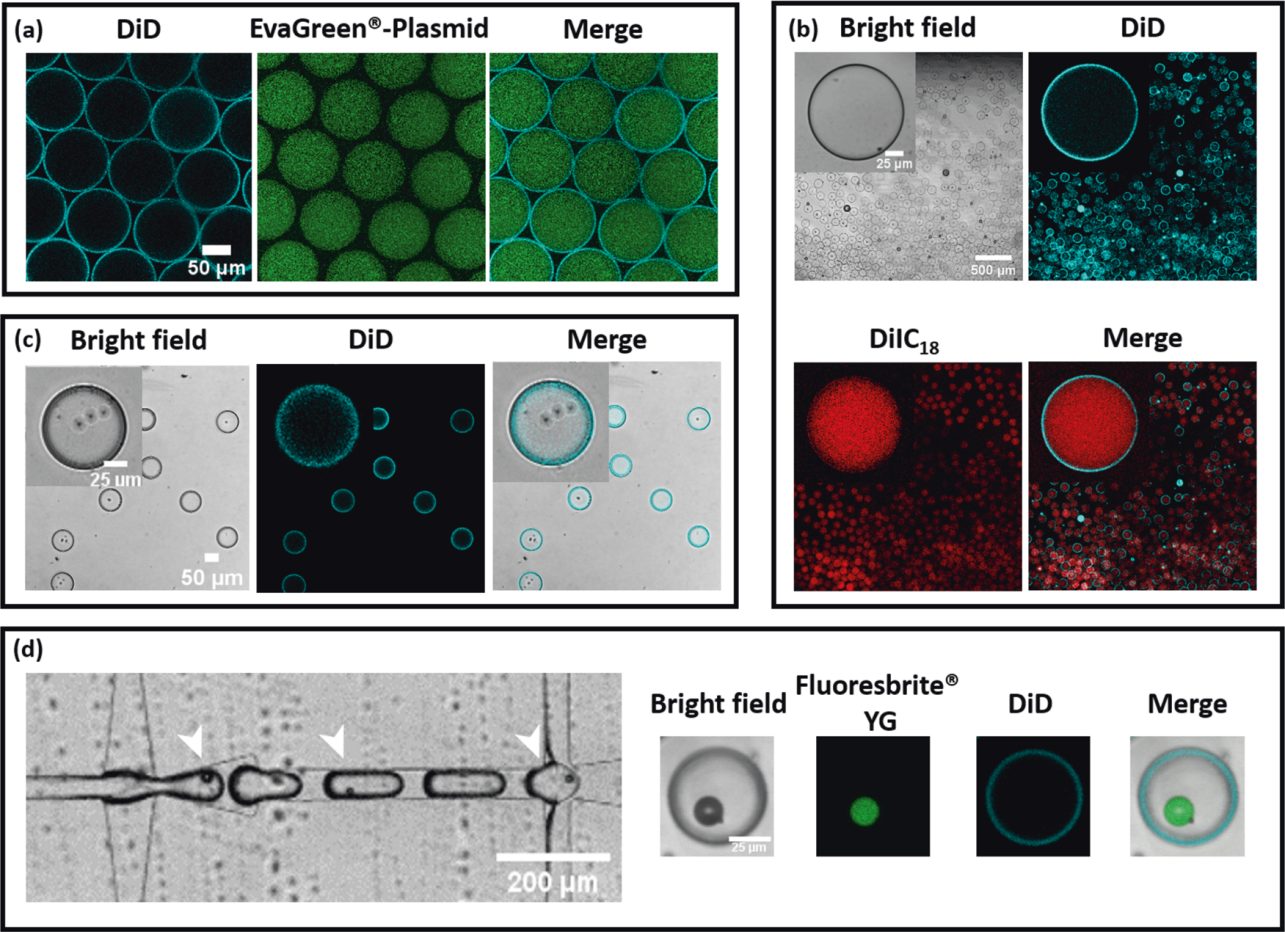
Microfluidic production of artificial cells. **a** Encapsulating EvaGreen^®^-plasmid DNA, **b** showing compartmentalization with SUVs, **c** encapsulating fibroblast cells along with the culture media, and **d** encapsulating microplastics such as styrene microspheres.

We have tested the applicability of the microfluidic device and also ascertained its robustness to produce artificial cells/carriers of various kinds. The possibility to incorporate large biomolecules is the major promise of using emulsion-based methods and microfluidics for their high-throughput capabilities. In Fig. 5, experiments were conducted to prove this by incorporating a range of large molecules from plasmid DNA to microspheres. Firstly, as a proof-of-concept, IA containing circular plasmid DNA with EvaGreen® dye was used to make liposomes with water as the OA (Fig. 5a and Supplementary Movie 3). The produced liposomes are not only homogeneous in size, but also have uniform green fluorescence intensity across all of the analyzed luminal cross-sections and not on the lipid membrane (Supplementary Fig. 4). The uniformity shown here proves that the lipid membrane and the lumen of the vesicle are free of oil residues. This result is a step toward building an artificial cell—by adding ingredients required for translation and transcription like in prokaryotic cells. As a follow-up to the concept of constructing artificial cells, we have incorporated HEPES buffer containing small unilamellar vesicles (SUVs) inside these giant liposomes to mimic eukaryotic cell architecture (Fig. 5b). SUVs are highly robust small vesicles in the range of 50 nm in diameter. Protocols for producing and encapsulating desired materials inside these SUVs and even LUVs (large unilamellar vesicles) are very well established^2^. We present the microfluidic device and the associated result (Fig. 5b) as a potential way to study the evolution of prokaryotic cells to eukaryotic cells, more specifically compartmentalization and their role in organized decentralization within cells. Note that the high-throughput nature of the microfluidics is evident from the large populations of liposomes that can be seen in Fig. 5b and the robustness of the technique in using HEPES or other buffered solutions with ease, unlike standard techniques like electroformation. A step further is to encapsulate cells within the liposomes (Fig. 5c). Lipid-based vesicles present a natural environment for the cellular growth, considering that cells interact with other cells in tissues or biofilms. Recently, biofilms have been made within droplets that can be used for understanding biofilm growth, expansion, and even high-throughput screening^38,39^. Lipid vesicles that can be produced with ease using PDMS-based microfluidics will be a boon to either single-cell studies or even the development of organoids and biofilms for drug discovery. PDMS-based devices provide an additional advantage as the produced vesicles can be subsequently captured and analyzed in the same device^40^. The data in Fig. 5c demonstrates the ability to encapsulate fibroblast cells using this device (~75% of them containing cells). One other interesting aspect of utilizing microfluidics to produce lipid vesicles is the possibility to incorporate very large molecules, even nonbiological foreign bodies. In Fig. 5d, we incorporated large styrene microspheres of ∼20 µm diameter inside the microfluidic GUVs. While it was not possible to incorporate microspheres inside every liposome, due to not being dispersible in aqueous solutions unlike the above-mentioned examples (plasmid, liposomes, and cells), approximately 30% contained microspheres. While this can be considered as a potential limitation, it would be possible to add a packing module to future designs to improve the encapsulation for non-dispersible components. Considering the high-throughput nature of the production process, this encapsulation rate provides a high number to study the interaction between non-degradable, environmentally toxic materials and cell membranes (Supplementary Movie 4). We note that for dispersible components, a high fluorescence encapsulation efficiency is observed (~96% for EvaGreen^®^-plasmid DNA and ~94% for SUVs).

## Discussion

Despite many groups using microfluidics to produce artificial cells in recent years, the issue of how biomimetic they are still remains. This can either be due to the continued use of non-biocompatible surfactants or reagents to form stable vesicles or because residual oil in the membrane is largely ignored. Here, we not only produce stable mono-disperse GUVs at high-throughput, but this method does not require these unwanted additives. Furthermore, our study is supported by a thorough analysis of the remaining non-detectable oil. While some studies do not require a pure-lipid membrane, many do and until now researchers were limited to swelling-based methods.

A popular choice of microfluidics in this field is the use of glass microcapillaries, and while they are harder to assemble than the PDMS-based alternatives as they require precise alignment steps, the coating of each separate channel is easier. Our approach was to use PDMS, as the assembly is straightforward, but this also required us to greatly simplify the pre-coating steps for ease-of-use: finally taking only 5 min which allows more experimental flexibility and device reliability. Note that previous PDMS-based methods use elevated temperatures, lengthy incubation times, or multiple syringe pumps to pre-coat the channels^14,16,32–34^. These are often large barriers to non-microfluidic groups wanting to adopt these advanced techniques. In this work, only a simple vacuum pump was employed to draw the solutions into the channels one after the other as explained in the methods. However, it is important to acknowledge that the coating process adopted here is prone to instabilities at pH conditions below 5 and above 10 due to its polyelectrolyte nature. Furthermore, it is also advisable to first flush the OA into the chip before introducing the LO as mentioned in the “Results” section—this is only a precautionary step to prevent any possible erosion of the coating if any. We also note that PDMS-based platforms are inherently less solvent compatible compared to glass ones.

Compared to other methods, both capillary- and PDMS-based, we report greater control over the size of the vesicles in the form of an extended range of GUV diameters: from ∼10 to 130 µm^14,24^. This is a particular advantage as a single device can be used to encapsulate the same reactants to explore surface-to-volume effects for example. This is due to a high level of control offered by our microfluidic design, i.e., over the size of the W/O droplets formed at the first junction and W/O/W emulsions at the second junction. Such a double junction device offers advanced control and does not need viscosity enhancers like PVA or glycerol that were needed in the case of single-junction devices reported elsewhere^14^. Furthermore, the delay between the junctions, the concentration of lipids, and assisted advective transport of lipids to the interface at the constriction sites promotes faster assembly and removes the need for surfactants such as block co-polymers. The gradual de-wetting of the oil phase in association with zipping of the lipids yields lipid membranes that are stable enough to produce vesicles. This mechanism is similar to that of other microfluidic double emulsion methods but is not the case for bulk and on-chip phase transfer methods (i.e., cDICE) where the need for lipid monolayer re-sealing hinders high throughput production^9,41^. Another degree of freedom reported here is the ability to produce vesicles from charged lipids, free of contamination from surfactants or oil. Our methodology now allows researchers to build artificial cells with biomimetic charged lipid mixtures and with the encapsulated machinery to mimic cell-like processes or to study protein–membrane interactions. Compared to other methods, this work offers a greater range of buffers from salt-based to pure water—until now solution additives rendered both bulk and microfluidic GUVs nonphysiological.

Compared to bulk methods (swelling or inverted emulsions), few groups have combined the control of microfluidic lipid vesicle production with encapsulating large biomolecules^18,41^. In this work, we have not only shown the versatility of the microfluidic device to produce complex configurations of artificial cells in a single platform for the first time but also with high encapsulation efficiencies for dispersible biomolecules while still retaining the biomimetic nature of the vesicles produced. Encapsulating biological cells within GUVs either for bioanalytical purposes or as functional modules has yet to be fully explored perhaps owing to the continued general use of non-biocompatible additives. We, therefore, expect others to adopt our approach to advance this promising new field. Similarly, others have reported issues with protein aggregation^25^ or membrane rupture^25,42^ in the presence of PVA or glycerol respectively. However, this problem is overlooked with emulsion-based methods where these are commonly needed to produce membranes in the presence of proteins. As cell-mimicking systems become increasingly more complex, this issue which lowers yields and could compromise enzyme activity, cannot be overlooked. Therefore, our method will hopefully form the basis of successful future endeavors in bottom-up synthetic biology especially to produce asymmetric bilayers that are more true to eukaryotic cells. Finally, we note the robustness of this design as stable double emulsions can be continuously produced without collapse or blockages for at least as long as the fluid in the reservoirs lasts. An advantage that also applies to applications outside of artificial cells where reactions are initiated within double emulsions in general. The promise of high-throughput emulsion-based technologies is immense and the microfluidic device design and methodologies employed here will help progress the field of artificial cells considerably in the near future.

## Methods

### Materials

All materials were used as purchased unless noted otherwise. 1-octanol (99%, Sigma Aldrich), (97%, Sigma Aldrich). 1-Palmitoyl-2-oleoyl-sn-glycero-3-phosphocholine (POPC), 1,2-dioleoyl-sn-glycero-3-phospho-L-serine (DOPS) and 1,2-dioleoyl-sn-glycero-3-phosphoethanolamine-N-(lissamine rhodamine B sulfonyl) (ammonium salt) (Liss Rhod DOPE). was obtained from Avanti polar lipids. 1,1′-Dioctadecyl-3,3,3′,3′-tetramethylindotricarbocyanine perchlorate (DiIC_18_ (3)) and DiD (DiIC_18_ (5)) and calcein were purchased from ThermoFisher Scientific. Polydimethylsiloxane (PDMS) and curing agent were obtained as SYLGARD®184 silicone elastomer kit from Dow Corning. 1H,1H,2H,2H-Perfluorodecyltrichlorosilane was purchased from abcr GmbH. Poly(diallyldimethylammonium) chloride (PDADMAC) and poly(sodium 4-styrenesulfonate) (PSS) were obtained from Sigma Aldrich. SU8 2050 (Microchem Inc.), Silicon wafer (Siegert Wafers), SU8 developer solution (Microchem Inc.). Indium tin oxide (ITO) glass slides are from Präzisions Glas & Optik GmbH. Glucose, sucrose, and chloroform were obtained from Merck. HEPES and PBS were purchased from Sigma-Aldrich. Dulbecco’s Modified Eagle Medium (DMEM) was purchased from ThermoFisher Scientific. Mini Extruder and polycarbonate membranes were purchased from Avanti polar lipids, Inc. Fluoresbrite^®^ YG Microspheres purchased from Polysciences, Inc. Microfluidic lipid vesicle production was achieved using an MFCS^™^-EX pressure pump with associated 2 mL reservoirs for various solutions from Fluigent, Inc.

### Microfluidic device fabrication

The PMDS-based microfluidic device fabrication was performed using soft-photolithography as described previously^8^. Master molds were prepared on 4′′ silicon wafers using a spin coating (model no. WS-650MZ-23NPPB, Laurell Tech. Corp.) SU8 2025 (Microchem Inc.) to a height of 80 μm. Following the coating process, a pre-baking step was performed before UV-light exposure through a film mask with the requisite design (see Supplementary Data 1 for the CAD file), onto the SU8 coated silicon wafer for a duration of 8 s (MicroLithography Services). A post-baking step was performed before the SU8 development process. SU8 development was performed by gently washing the wafer in developer solution (Microchem Inc.) for 3 min. Finally, the Si-wafer was hard-baked for a period of 30 min at 200 °C. Then the prepared master molds were silanized overnight (50 μl of 1H,1H,2H,2H-perfluorodecyltrichlorosilane) in a desiccator. PDMS-based microfluidic chips were produced by heat curing (90 °C for 3 h) PDMS with curing agent mixture (10:1) on the master mold Si-wafer. Cured PDMS was peeled and cut into individual chips. Holes were punched at respective inlet and outlets using a 1 mm biopsy puncher (Kai Europe GmbH) before bonding the PDMS to glass coverslips. At the end, bonding was performed using an air plasma treatment (Plasma Cleaner PDC-002-CE, Harrick Plasma) at 600 mbar for 1 min. The microfluidic devices were heated for 2 h at 60 °C to help with the bonding process and render the PDMS back to hydrophobic after the plasma treatment. A photograph of a final assembled device can be seen in Supplementary Fig. 1a.

### Surface coating of the microchannels

W/O/W emulsion production requires the surface of the outer channel (from OA inlet to outlet, see Fig. 1b in the main text) to be hydrophilic. Unless the outer channel is wettable by an aqueous medium, the W/O/W type of double emulsion will not form. This is because the material used in this study to make the microfluidic chip, PDMS, is hydrophobic in nature and is not wettable by an aqueous medium. For the outer aqueous solution (OA) to be able to wet the PDMS surface, the channel was treated with series of chemical reagents that are drawn into the microfluidic chip using a vacuum pump. Channels carrying OA toward the outlet (see Fig. 1b) were subjected to an initial cleaning step by flushing HCl:H_2_O_2_ (1:2) for 30 s. This renders the chip surface negatively charged for the polymeric solution of positively charged polymer (2 wt.% PDADMAC) to form layers after flushing the solution for 2 min. Following this, a negatively charged polymeric solution (5 wt% PSS) was flushed for the same duration to yield a hydrophilic OA outlet channel. After every step, mention above, MilliQ^®^ water was flushed in for a minimum of 30 s to remove excess chemical reagents. Thus coated channels remained hydrophilic for more than a week and can remain functional during experimentation for more than 5 h. Importantly, the entire coating process takes up to only 5 min to complete and the device is ready to use thereafter. In the case of sub-optimal coating, higher concentrations of the polymer solutions or longer coating procedures can be employed.

### Liposome production: microfluidics, electroformation, and extrusion

A double cross-junction chip design was used to produce the double emulsions (to eventually form GUVs). To make W/O/W double emulsion, the IA solution containing MilliQ^®^, EvaGreen^®^-plasmid DNA (equimolar mixture), calcein, SUVs, cells or styrene microspheres was passed through the first cross-junction to be sheared into aqueous droplets by 1-octanol with 5 mg/mL total lipid concentration of 99.5 mol% POPC and 0.5 mol% DiD/DiIC_18_/Liss Rhod PE). Following this, the oil phase (LO) carrying aqueous droplets was further sheared to produce double emulsion at the second cross-junction by the outer aqueous (OA) solution of MilliQ^®^ water/300 mOsm glucose solution/HEPES buffer (20 mM). Electroformed GUVs were produced using ITO-coated glass plates. 15 µL of 2 mg/mL total lipid concentration of 99.5 mol% POPC and 0.5 mol% Liss Rhod PE was smeared over the ITO surface side of the glass plates and dried using a nitrogen gun and further in a desiccator at low-pressure conditions for 45 min. A chamber was created with both the lipid-coated surfaces facing inwards using a Teflon spacer. This chamber was filled with 300 mOsm sucrose solution and sealed. Electric input was delivered to the ITO sides of the glass plates through copper tapes connected to a function generator at 10 Hz AC and 2 Vp-p for 2 h. After the production, glass plates were finger-tapped for the release of the GUVs into the sucrose solution before they are collected in an Eppendorf^®^ tube. The 50 nm size SUVs were produced using the hydration and extrusion method. Totally, 24 mM total lipid concentration of 99.5 mol% POPC and 0.5 mol% DiIC_18_ in chloroform were dried onto a glass vial using a nitrogen gun and in a desiccator at low-pressure conditions for 45 min. This was followed by hydration of the lipid layer with 1 mL of warm HEPES buffer (37 °C). Hydration was allowed to take place overnight at room temperature. The produced multilamellar vesicles (MLVs) were subjected to freeze–thaw cycle using liquid nitrogen and a hot water bath. Further to this, MLVs were made into SUVs using a lipid extruder fitted with a 50 nm polymeric filter. After 21 extrusion cycles, the SUVs produced were used in microfluidic production to make compartmentalized liposomes.

### Cell culture

Fibroblast cells were cultured in DMEM media at 37 °C and retrieved using trypsin enzyme from Petri dishes. The suspension is directly used as the IA to therefore demonstrate the possibility to encapsulate cells within liposomes produced using microfluidics.

### Fluorescence recovery after photobleaching (FRAP)

The fluorescence image sequences of the liposomes were acquired using the 488 nm line of an argon ion laser at a very low power to avoid photobleaching. After 2.5 s, regions of interest (ROI), of 6 μm radius on the top surface of the vesicles were rapidly photobleached (*t* < 60 ms) at maximal laser power. Fluorescence recovery was monitored for ~20 s. The recovery curves were obtained as explained in a previous work^43^. In order to correctly estimate *F*_0_ (the fluorescence intensity immediately after the end of the bleach) and *F*_18s_ (after 18 s), the curves were fitted as in ref. ^43^. The normalized fractional recovery (NFR) is defined as

$${\rm{NFR}} = \frac{{F_{18s} - F_0}}{{1 - F_0}}$$

Following this, a nonlinear exponential curve fitting was performed (OriginPro, OriginLab Corp.) on the fractional recovery curve from each data set to calculate the halftime (*t*_half_) of recovery. Using the *t*_half_ obtained, the diffusion coefficient for the lipid DOPE-Liss Rhod was determined using

$$D = 0.25\omega ^2{\mathrm{/}}t_{\rm{half}}$$

where *D* is the diffusion coefficient and *ω* is the radius of the bleach spot.

### Dye-leakage assay

The leakage of calcein dye from the lipid bilayer has been used in earlier works as a tool to investigate the functionality and unilamellar of model lipid membranes^8,14^. We have tested the liposomes produced in this work for leakage both in the presence and absence of α-hemolysin as a pore-forming protein. Liposomal vesicles were produced with 20 µM calcein solution as the IA. To which, 2.5 µg/mL final concentration of α-hemolysin was added. The graph shown in Fig. 4c presents the recording obtained after 10 min of incubation.

### Microscopy

The produced liposomes which were collected in Eppendorf^®^ tubes were pipetted onto BSA coated (2 mg/mL for 30 min at 37 °C) glass coverslips and sealed using SecureSeal^™^ image spacers (Sigma Aldrich). A MicroLab 310 (Vision Research Inc.) high-speed camera fitted to an Olympus IX73 microscope was used to acquire images of the liposome production at full-frame and with ~3000 frame rate. Liposomes produced were also visualized using confocal microscopy (Leica TCS SP8, Leica Microsystems Inc.). For image acquisition, ex488/em499–540 nm for EvaGreen^®^, Fluoresbrite® Yellow Green (YG), and calcein, ex551/em565–610 nm for DiIC_18_, and Liss Rhod DOPE and ex633/em645–680 nm for DiD wavelengths were used. Data produced were treated and analyzed using ImageJ software.

### Computational fluid dynamics

To prove that constricted curves can result in increased forces, a computational fluid dynamics simulation was performed using FEATool Multiphysics for MATLAB^®^. Using a 2D replication of the design used in this work, the first constriction of the serpentine channel region was drawn with the built-in geometry tools. Following this, a 2D grid was generated and refined using the Gmsh mesh builder. The Navier–Stokes equation (for incompressible fluids) was used to simulate the fluid flow.

## Supplementary information

Supplementary Information

Description of Additional Supplementary Files

Supplementary Data 1

Supplementary Movie 1

Supplementary Movie 2

Supplementary Movie 3

Supplementary Movie 4

## Data Availability

Supplementary Information is provided together with Supplementary Data 1 and Supplementary Movies 1–4. All other relevant data are available from the corresponding author.

## References

[1] Hernandez, E. M. Pharmaceutical and cosmetic use of lipids. *Bailey’s Ind. Oil Fat Prod*. 10.1002/047167849x.bio068.pub2 (2020).

[2] Sercombe, L. et al. Advances and challenges of liposome assisted drug delivery. *Front. Pharmacol.*10.3389/fphar.2015.00286 (2015). 10.3389/fphar.2015.00286 PMC4664963 26648870

[3] Mutschler, H., Robinson, T., Tang, T. Y. D. & Wegner, S. Special issue on bottom-up synthetic biology. *ChemBioChem*10.1002/cbic.201900507 (2019). 10.1002/cbic.201900507 31573136

[4] Schwille, P. et al. MaxSynBio: avenues towards creating cells from the bottom up. *Angew. Chem.*10.1002/anie.201802288 (2018). 10.1002/anie.201802288 29749673

[5] Robinson, T. Microfluidic handling and analysis of giant vesicles for use as artificial cells: a review. *Adv. Biosyst.*10.1002/adbi.201800318 (2019). 10.1002/adbi.201800318 32648705

[6] *The Giant Vesicle Book*. *The Giant Vesicle Book*10.1201/9781315152516 (2019).

[7] Weinberger A (2013). Gel-assisted formation of giant unilamellar vesicles. Biophys. J..

[8] Yandrapalli N, Robinson T (2019). Ultra-high capacity microfluidic trapping of giant vesicles for high-throughput membrane studies. Lab Chip.

[9] Moga A, Yandrapalli N, Dimova R, Robinson T (2019). Optimization of the inverted emulsion method for high-yield production of biomimetic giant unilamellar vesicles. ChemBioChem.

[10] Göpfrich, K. et al. One-pot assembly of complex giant unilamellar vesicle-based synthetic cells. *ACS Synth. Biol*. 10.1021/acssynbio.9b00034 (2019). 10.1021/acssynbio.9b00034 PMC6528161 31042361

[11] Hindley, J. W. et al. Building a synthetic mechanosensitive signaling pathway in compartmentalized artificial cells. *Proc. Natl. Acad. Sci. USA*10.1073/pnas.1903500116 (2019). 10.1073/pnas.1903500116 PMC6708380 31371493

[12] Hadorn M, Boenzli E, Eggenberger Hotz P, Hanczyc MM (2012). Hierarchical unilamellar vesicles of controlled compositional heterogeneity. PLoS ONE.

[13] Pautot, S., Frisken, B. J. & Weitz, D. A. Production of unilamellar vesicles using an inverted emulsion. *Langmuir*10.1021/la026100v (2003).

[14] Deshpande, S., Caspi, Y., Meijering, A. E. C. & Dekker, C. Octanol-assisted liposome assembly on chip. *Nat. Commun*. 10.1038/ncomms10447 (2016). 10.1038/ncomms10447 PMC4735860 26794442

[15] Shum, C. H. et al. Double Emulsion Templated Monodisperse Phospholipid Vesicles. *Langmuir*10.1021/la801833a (2008). 10.1021/la801833a 18613709

[16] Krafft, D. et al. Compartments for synthetic cells: osmotically assisted separation of oil from double emulsions in a microfluidic chip. *ChemBioChem*10.1002/cbic.201900152 (2019). 10.1002/cbic.201900152 PMC6852271 31090995

[17] Teh, S. Y., Khnouf, R., Fan, H. & Lee, A. P. Stable, biocompatible lipid vesicle generation by solvent extraction-based droplet microfluidics. *Biomicrofluidics*10.1063/1.3665221 (2011). 10.1063/1.3665221 PMC3368830 22685501

[18] Weiss, M. et al. Sequential bottom-up assembly of mechanically stabilized synthetic cells by microfluidics. *Nat. Mater*. 10.1038/NMAT5005 (2018). 10.1038/nmat5005 29035355

[19] Petit, J., Polenz, I., Baret, J. C., Herminghaus, S. & Bäumchen, O. Vesicles-on-a-chip: a universal microfluidic platform for the assembly of liposomes and polymersomes. *Eur. Phys. J. E*10.1140/epje/i2016-16059-8 (2016). 10.1140/epje/i2016-16059-8 27286954

[20] Hezaveh, S., Samanta, S., De Nicola, A., Milano, G. & Roccatano, D. Understanding the interaction of block copolymers with DMPC lipid bilayer using coarse-grained molecular dynamics simulations. *J. Phys. Chem. B*10.1021/jp306565e (2012). 10.1021/jp306565e 23137298

[21] Zhang, W. et al. Quantifying binding of ethylene oxide-propylene oxide block copolymers with lipid bilayers. *Langmuir*10.1021/acs.langmuir.7b02279 (2017). 10.1021/acs.langmuir.7b02279 PMC6055234 29068209

[22] Maskarinec, S. A., Hannig, J., Lee, R. C. & Lee, K. Y. C. Direct observation of poloxamer 188 insertion into lipid monolayers. *Biophys. J*. 10.1016/S0006-3495(02)75499-4 (2002). 10.1016/S0006-3495(02)75499-4 PMC1301946 11867460

[23] Alakhova DY, Kabanov AV (2014). Pluronics and MDR reversal: an update. Mol. Pharm..

[24] Deng, N. N., Yelleswarapu, M. & Huck, W. T. S. Monodisperse uni- and multicompartment liposomes. *J. Am. Chem. Soc*. 10.1021/jacs.6b02107 (2016). 10.1021/jacs.6b02107 27243596

[25] Abou-Saleh, R. H. et al. Molecular effects of glycerol on lipid monolayers at the gas-liquid interface: impact on microbubble physical and mechanical properties. *Langmuir*10.1021/acs.langmuir.8b04130 (2019). 10.1021/acs.langmuir.8b04130 30901226

[26] Schaich, M., Sobota, D., Sleath, H., Cama, J. & Keyser, U. F. Characterization of lipid composition and diffusivity in OLA generated vesicles. *Biochim. Biophys. Acta*. 10.1016/j.bbamem.2020.183359 (2020). 10.1016/j.bbamem.2020.183359 PMC7322398 32416194

[27] Ho, K. K. Y., Lee, J. W., Durand, G., Majumder, S. & Liu, A. P. Protein aggregation with poly(vinyl) alcohol surfactant reduces double emulsionencapsulated mammalian cell-free expression. *PLoS ONE*10.1371/journal.pone.0174689 (2017). 10.1371/journal.pone.0174689 PMC5373588 28358875

[28] Dao, T. P. T. et al. Membrane properties of giant polymer and lipid vesicles obtained by electroformation and pva gel-assisted hydration methods. *Colloids Surfaces A Physicochem. Eng. Asp*. 10.1016/j.colsurfa.2017.09.005 (2017).

[29] Machta, B. B. et al. Conditions that stabilize membrane domains also antagonize n-alcohol anesthesia. *Biophys. J*. 10.1016/j.bpj.2016.06.039 (2016). 10.1016/j.bpj.2016.06.039 PMC4982967 27508437

[30] Thutupalli, S., Fleury, J. B., Steinberger, A., Herminghaus, S. & Seemann, R. Why can artificial membranes be fabricated so rapidly in microfluidics? *Chem. Commun*. 10.1039/c2cc38867g (2013). 10.1039/c2cc38867g 23321691

[31] Arriaga, L. R., Amstad, E. & Weitz, D. A. Scalable single-step microfluidic production of single-core double emulsions with ultra-thin shells. *Lab Chip*10.1039/c5lc00631g (2015). 10.1039/c5lc00631g 26152396

[32] Lu, L., Schertzer, J. W. & Chiarot, P. R. Continuous microfluidic fabrication of synthetic asymmetric vesicles. *Lab Chip*10.1039/c5lc00520e (2015). 10.1039/c5lc00520e 26220822

[33] Karamdad K, Law RV, Seddon JM, Brooks NJ, Ces O (2016). Studying the effects of asymmetry on the bending rigidity of lipid membranes formed by microfluidics. Chem. Commun..

[34] Trantidou, T., Elani, Y., Parsons, E. & Ces, O. Hydrophilic surface modification of pdms for droplet microfluidics using a simple, quick, and robust method via pva deposition. *Microsyst. Nanoeng*. 10.1038/micronano.2016.91 (2017). 10.1038/micronano.2016.91 PMC6444978 31057854

[35] Lindblom, G. & Orädd, G. Lipid lateral diffusion and membrane heterogeneity. *Biochim. Biophys. Acta*10.1016/j.bbamem.2008.08.016 (2009). 10.1016/j.bbamem.2008.08.016 18805393

[36] Toppozini, L. et al. Partitioning of ethanol into lipid membranes and its effect on fluidity and permeability as seen by X-ray and neutron scattering. *Soft Matter*10.1039/c2sm26546j (2012).

[37] Pincet F (2016). FRAP to characterize molecular diffusion and interaction in various membrane environments. PLoS ONE.

[38] Jin, Z. et al. Dynamic sessile-droplet habitats for controllable cultivation of bacterial biofilm. *Small*10.1002/smll.201800658 (2018). 10.1002/smll.201800658 29717806

[39] Chang, C. B., Wilking, J. N., Kim, S. H., Shum, H. C. & Weitz, D. A. Monodisperse emulsion drop microenvironments for bacterial biofilm growth. *Small*10.1002/smll.201403125 (2015). 10.1002/smll.201403125 25959709

[40] Yandrapalli, N., Seemann, T. & Robinson, T. On-chip inverted emulsion method for fast giant vesicle production, handling, and analysis. *Micromachines*10.3390/mi11030285 (2020). 10.3390/mi11030285 PMC7142477 32164221

[41] Abkarian, M., Loiseau, E. & Massiera, G. Continuous droplet interface crossing encapsulation (cDICE) for high throughput monodisperse vesicle design. *Soft Matter*10.1039/c1sm05239j (2011).

[42] Pocivavsek, L. et al. Glycerol-induced membrane stiffening: the role of viscous fluid adlayers. *Biophys. J*. 10.1016/j.bpj.2011.05.036 (2011). 10.1016/j.bpj.2011.05.036 PMC3127174 21723821

[43] Escoffre, J. M., Hubert, M., Teissié, J., Rols, M. P. & Favard, C. Evidence for electro-induced membrane defects assessed by lateral mobility measurement of a gpi anchored protein. *Eur. Biophys. J*. 10.1007/s00249-014-0961-1 (2014). 10.1007/s00249-014-0961-1 24781652

