# Supplementary material for: Surfactant-free production of biomimetic giant unilamellar vesicles using PDMS-based microfluidics
Source: Commun Chem. 2021 Jun 29;4:100. doi: 10.1038/s42004-021-00530-1 (PMC9814093; doi:10.1038/s42004-021-00530-1)
Supplement: Supplementary file 1 — Supplementary Information [file 42004_2021_530_MOESM1_ESM.pdf]

Supporting Information

for

Surfactant-free production of biomimetic giant unilamellar vesicles using PDMS-based microfluidics

Naresh Yandrapalli,[1] *Julien Petit,*[2] *Oliver Bäumchen,*[2,3] *and Tom Robinson*[1]*

[1]Max Planck Institute of Colloids and Interfaces, Department of Theory & Bio-Systems, Am Mühlenberg 1, 14424 Potsdam, Germany

[2]Max Planck Institute for Dynamics and Self-Organization, Am Faßberg 17, 37077 Göttingen, Germany

[3]Experimental Physics V, University of Bayreuth, Universitätsstr. 30, 95447 Bayreuth, Germany

*E-mail: tom.robinson@mpikg.mpg.de

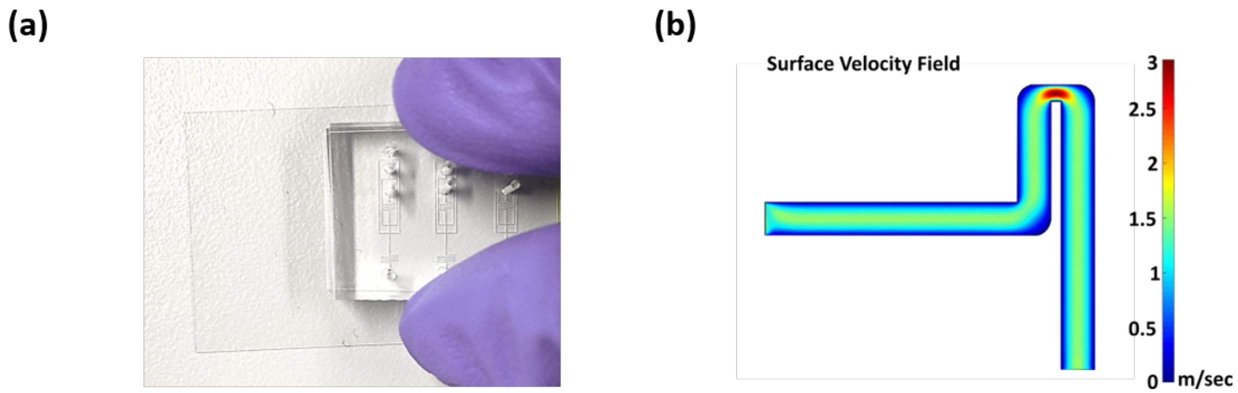

Supplementary Figure 1. Microfluidic Device and Flow simulation. (a) Photograph showing the final fabricated microfluidic chip bonded to a glass coverslip. Each chip has six copies of the design for repeat experiments and convenience. (b) CFD simulation in 2D of the first constriction of the serpentine module present in our microfluidic design.

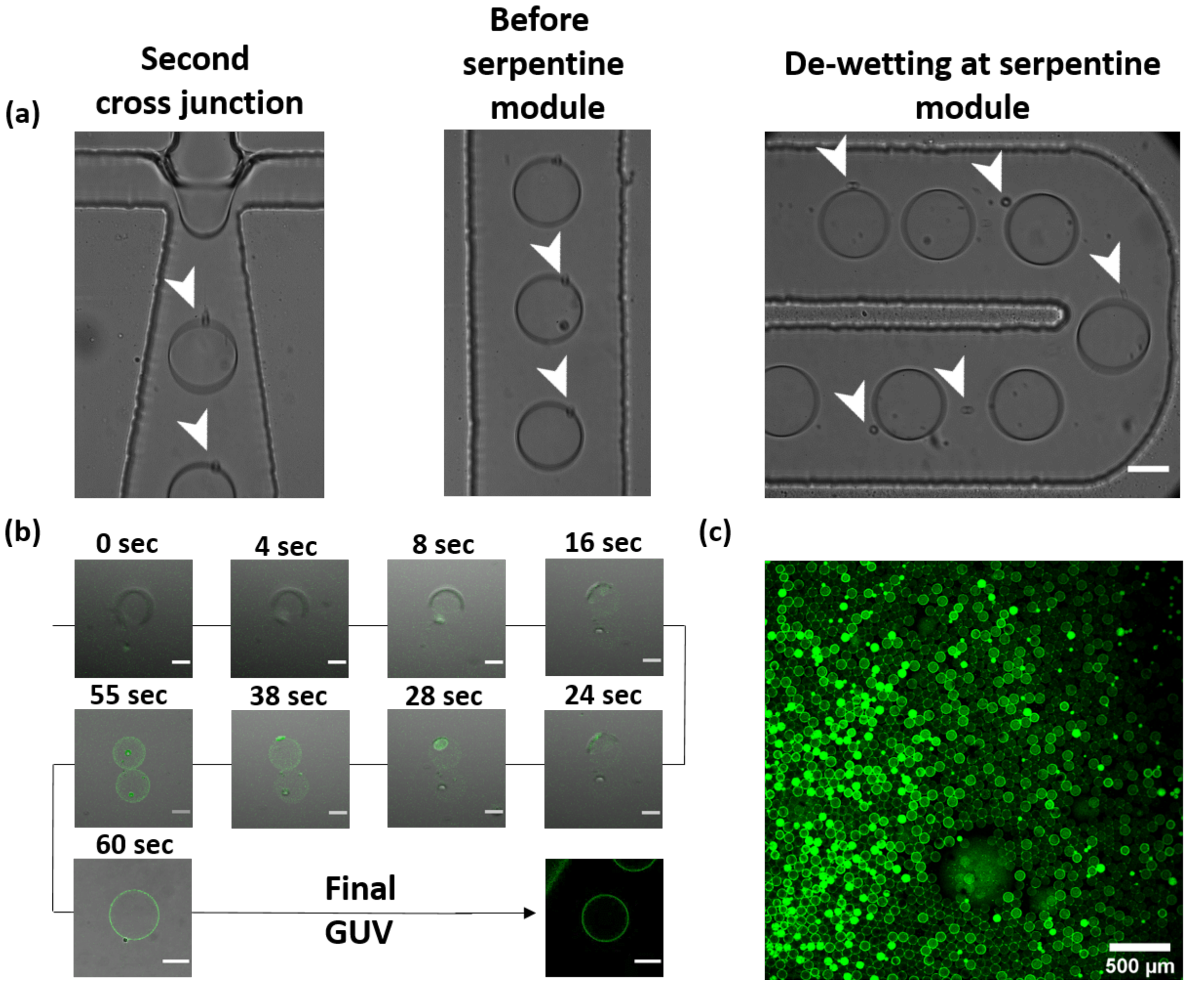

Supplementary Figure 2. Dewetting process to form microfluidic lipid vesicles. (a) Snapshots of vesicles forming in the microfluidic channels, starting with the formation of double emulsion at the second junction and spontaneous 1-octanol dewetting and pinch-off - enhanced by the serpentine module (arrows showing the oil droplets). Scale bar corresponds to 50 µm). (b) Snapshots of the left-over oil residues, in some cases, dewetting and pinching-off from individual vesicles in the solution. Scale bar corresponds to 50 µm. (c) Wide field of view (x5 objective) of large number of vesicles produced using the microfluidic device along with the dewetted oil droplets (bright green spots).

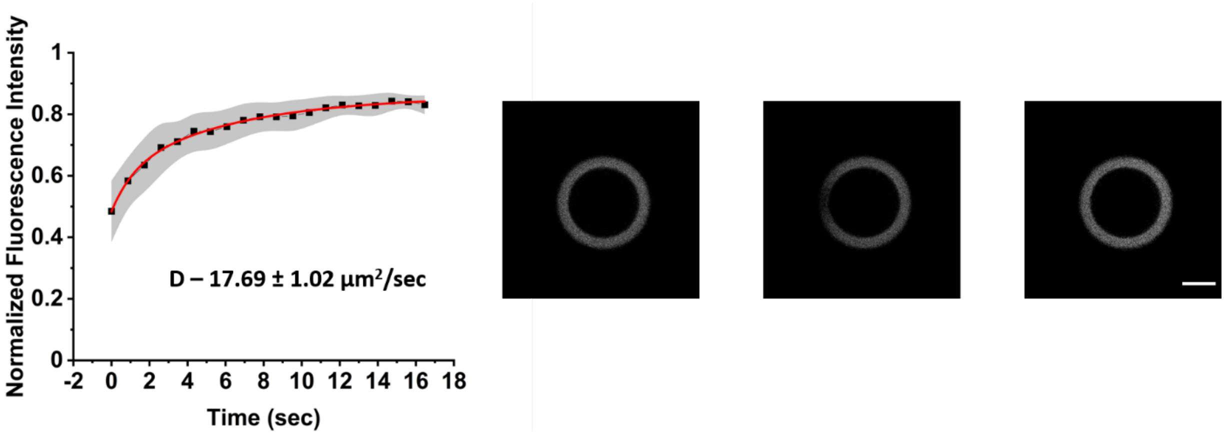

Supplementary Figure 3. Fluorescence Recovery After Photobleaching (FRAP) measurements on double emulsions. Left: average fluorescence recovery curve of 7 double emulsions with visible oil layers produced using pressures of IA 52, LO 47, and OA 55 mbar. Error bars are taken from the standard deviation of the mean. Right: confocal cross-sections before (-2.5 s), during, and after (18 s) photobleaching. Note that photobleaching had to be performed on the sides of the double emulsions where the oil layer was thick and not on the top which was in contact with the coverslip due to buoyancy. Scale bar: 20 μm.

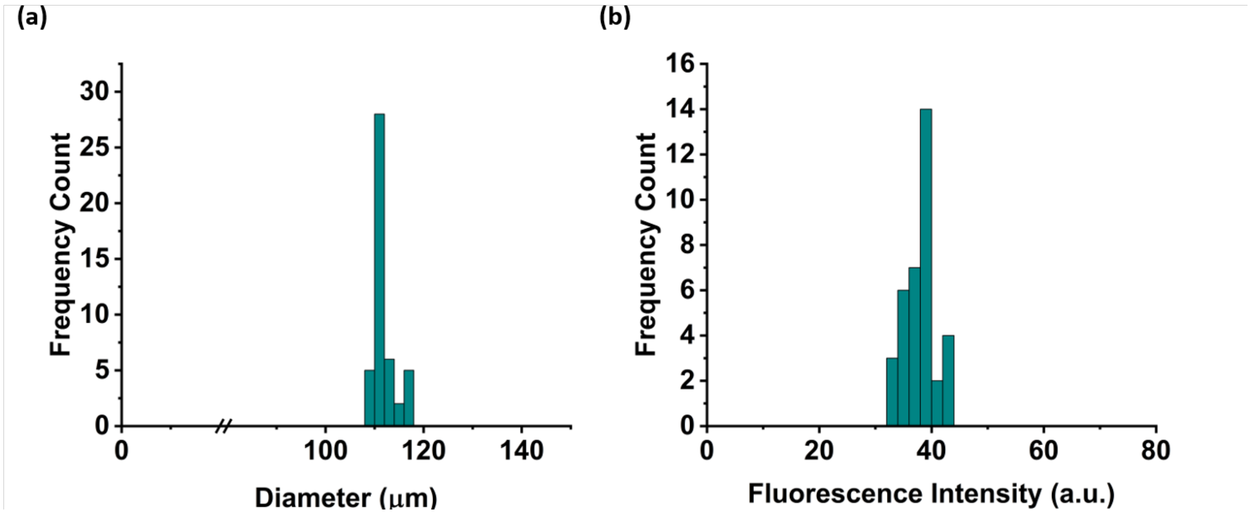

Supplementary Figure 4. Homogeneous size distribution of the liposomes produced by our microfluidic technique and its encapsulation efficiency. (a) The monodisperse nature of the microfluidic GUVs containing EvaGreen®-plasmid DNA, 110 ± 4 μm with RSD of 2.5% and (b) the EvaGreen®-plasmid DNA fluorescence intensity among various GUVs produced using this technique, showing a very narrow distribution.
