# Supplementary material for: Surfactant-free production of biomimetic giant unilamellar vesicles using PDMS-based microfluidics
Source: Commun Chem. 2021 Jun 29;4:100. doi: 10.1038/s42004-021-00530-1 (PMC9814093; doi:10.1038/s42004-021-00530-1)
Supplement: Supplementary file 2 — Description of Additional Supplementary Files [file 42004_2021_530_MOESM2_ESM.pdf]

Description of Additional Supplementary Files

File Name: Supplementary Movie 1

Description: Microfluidic production of liposomes with MilliQ® water in IA as well as in OA and POPC lipids in LO at 25 fps (IA-50 mbar, LO-44 mbar, and OA-57 mbar).

File Name: Supplementary Movie 2

Description: Video showing microfluidic production of liposomes. On the right-side liposomes with optically invisible oil layer (IA-52 mbar, LO-47 mbar, and OA-59 mbar) and the left-side with thick oil layer (IA-52 mbar, LO-47 mbar, and OA-51 mbar) produced by carefully altering the flow rates via pressure.

File Name: Supplementary Movie 3

Description: Microfluidic production of liposomes with EvaGreen®-plasmid DNA mix as IA (51 mbar), MilliQ® water as OA (100 mbar) and POPC lipids in LO (47 mbar).

File Name: Supplementary Movie 4

Description: Time-lapse video of styrene microsphere interaction with lipid membrane. At the end of the video it is evident that the microsphere has affinity towards the lipid membrane and resulted in binding.

File Name: Supplementary Data 1

Description: CAD file of the microfluidic design used in this work.
